# A new insight into the three-dimensional architecture of the Golgi complex: Characterization of unusual structures in epididymal principal cells

**DOI:** 10.1371/journal.pone.0185557

**Published:** 2017-09-28

**Authors:** Narcisa Martínez-Martínez, Emma Martínez-Alonso, Mónica Tomás, Josef Neumüller, Margit Pavelka, José A. Martínez-Menárguez

**Affiliations:** 1 Department of Cell Biology and Histology, Biomedical Research Institute of Murcia (IMIB-Arrixaca-UMU), University of Murcia, Murcia, Spain; 2 Memorial Sloan-Kettering Cancer Center, Howard Hughes Medical Institute, York Avenue, New York, New York, United States of America; 3 Department of Human Anatomy and Embryology, Medical School, Valencia University, Valencia, Spain; 4 Department of Cell and Developmental Biology, Center for Anatomy and Cell Biology, Medical University of Vienna, Schwarzspanierstrasse 17, Vienna, Austria; Chinese University of Hong Kong, HONG KONG

## Abstract

Principal epididymal cells have one of the largest and more developed Golgi complex of mammalian cells. In the present study, we have used this cell as model for the study of the three-dimensional architecture of the Golgi complex of highly secretory and endocytic cells. Electron tomography demonstrated the presence in this cell type of some unknown or very unusual Golgi structures such as branched cisternae, pocket-like cisternal invaginations or tubular connections. In addition, we have used this methodology and immunoelectron microscopy to analyze the close relationship between this organelle and both the endoplasmic reticulum and microtubules, and to describe in detail how these elements interact with compact and non-compact regions of the ribbon.

## Introduction

The Golgi complex (GC) is one of the most studied and photographed organelles and, conversely, it remains, perhaps, as the most mysterious one. Many open questions remain such as the mode of intra-Golgi transport and the exact role of tubules and vesicles (for review see references [1, 2, 3, 4, 5, 6, 7, 8]). In most text books the GC is drawn as a pile of a short number of flat cisternae surrounded by coated vesicles of different sizes, together with transitional elements of the endoplasmic reticulum (ER) and secretory granules at the cis and trans faces, respectively. Usually, some common Golgi characteristics, such as tubular networks connecting stacks or the so-called trans-ER, are not present in these drawings. Clearly, this classical view of the GC is an oversimplification, although useful for integrating the huge amount of morpho-functional data generated in the last decades. Electron tomography is a powerful tool for getting a deeper view of its architecture. The three-dimensional analysis has allowed visualizing, for instance, the variations between cisternae within the same stack, the nature of the carriers mediating ER-to-Golgi and post-Golgi transport and the close association of microtubules, ER and stack (for review see references [9,10, 11, 12, 13]). However, most of these studies on mammalian cells have been carried out in cell lines such as NRK, insulinoma, or hepatoma cells, which have relatively simple GCs. The analysis of highly developed GC of cells under native conditions may reveal unknown characteristics of this organelle.

The principal cells from epididymis have one of the most developed GCs known. The unique environment of the epididymis allows the sperm to acquire motility and the ability to fertilize the oocyte, a process named sperm maturation. Epididymal cells actively contribute to this process by secreting hundreds of proteins (for a review see reference [14]), and some of them are absorbed at the sperm surface. It explains the huge development of their GCs, especially in the proximal region of the organ (initial segment and caput). Proteins are also reabsorbed from the epididymal fluid which explains the high development of the endosomal/lysosomal machineries in the epididymal cells. The ultrastructure of principal cell GCs was extensively analyzed by Hermo’s group [15, 16]. Their beautiful images showed the elaborated organization of this organelle which is formed, as in other cell types, for stacks connected by dilated tubules, together with cis and trans tubular networks and different types of vesicles. The relation of these elements with the ER and microtubules was also described. However, 2D morphological analysis can give just a partial view of the complexity of this organelle and the relationship between all these elements. By using the advantages of electron tomography in combination with cryoimmunoelectron microscopy and classical transmission electron microscopic analyses, we re-examined the organization of the Golgi complex in this cell type as model of highly active cells in mammalian tissues. We provide structural evidence for the existence of new features of this organelle that have not been described before (branched cisterna), or are very rare (cisterna invaginations) or poorly analyzed (tubular connections). We also describe the close association of microtubules, ER and cisternae.

## Materials and methods

### Antibodies and reagents

Monoclonal antibodies against PDI, α-tubulin and GM130 were obtained from Enzo Life Sciences, Inc. (Farmingdale, NY 11735, USA), Sigma Aldrich (Barcelona, Spain), BD Transduccion Laboratories (Erembodegem, Belgium), respectively. Polyclonal antibody against Rab6 protein was from Santa Cruz Biotechnology Inc. (Santa Cruz, CA, USA). Protein A-gold was obtained from the department of Cell Biology at Utrecht University (Utrecht, The Netherlands).

### Animals

All animals (adult Sprague Dawley rats) used in this study were maintained on a 12h/dark/light cycle in the animal facility of the University of Murcia at a room temperature of 20°C and fed *ad libitum*. The procedures for animal use were approved by the Murcia University Ethics Committee and done in accordance with the ethical and legal standards of Spain mentioned in Royal Decree RDL 1201/2005 of 10th October on protection of animals, used for experimentation and other scientific proposes, and 2010/63/EU legislation on animal protection. All efforts were made to minimize the suffering of animals.

### Conventional electron microscopy

Five adult male Sprague-Dawley rats were sacrificed by overdose of CO_2_ in a closed chamber and the testes were immediately removed from the scrotum through the abdominal cavity. The initial segment of the epididymis was removed and cut into small 1mm^3^ pieces. The samples were washed in PBS and fixed in 2% glutaraldehyde in 0.2M sodium cacodylate buffer, pH7.4 for 2 h at 4ºC. After fixation, the tissue was post-fixed in potassium ferrocyanide reduced osmium tetroxide for 1h, dehydrated and embedded in Epon 812. Ultrathin sections were mounted onto formvar carbon-coated copper grids and contrasted with uranyl acetate and lead citrate. The ultrastructural studies were performed with a Jeol-1011 electron microscope.

### High-pressure freezing (HPF)-freeze substitution (FS)

For HPF-FS analyses, three adult male Sprague-Dawley rats (350-450g) were anesthetised with sodium pentobarbital. After anaesthesia, the epididymis was removed and cryoimmobilized using a Leica EM Pact high-pressure freezer (Leica, Vienna, Austria). Frozen samples were freeze-substituted in a Leica EM automatic freeze substitution (AFS) system (Leica, Vienna, Austria), where the substitution was performed in pure acetone containing 2% (wt/vol) osmium tetroxide and 0.1% (wt/vol) uranyl acetate at −90°C for 72 h. The temperature was gradually increased (5°C/h) to 4°C, held constant for 2 h, and then increased to room temperature and maintained for 1 h. Samples were washed for 1 h in acetone at room temperature and infiltrated in a graded series of Epon-acetone mixtures: 1:3 for 2 h, 2:2 for 2 h, 3:1 for 16 h, and pure Epon 812 (Ted Pella, Inc.) for 30 h. Samples were embedded in fresh Epon and polymerized at 60°C for 48 h.

### Electron tomography

300 nm thick sections were transferred to copper grids, and analyzed stained with uranyl acetate and lead citrate. Colloidal gold particles (10 nm) were then deposited on both surfaces of these sections for use as fiducial markers during subsequent image alignment. Single and dual axis tomography was performed in a Tecnai 20 transmission electron microscope (FEI Company, Eindhoven, The Netherlands) at 200 kV by acquisition of tilt series at a tilt range of ±65° with an increment of 1° using either a single high-tilt holder (FEI Company) or a high-tilt rotation holder (Gatan, Inc., Pleasanton, CA) and an EAGLE 4k CCD camera (FEI Company; chip size: 4,096 x 4,096 pixels). Tilt series data were digitally recorded automatically with the Xplore3D software (FEI Company) which allows compensating dislocations of the region of interest during tilting. The digital images were stored into stack files (*.mrc). Tilt series were aligned using 10 nm gold fiducials and reconstructed by means of the weighted back projection (WBP) algorithm using the IMOD software (Boulder Laboratory for 3D Electron Microscopy of Cells, University of Colorado, USA).

### Modeling

Membranes of the GC and other structures of interest were three-dimensionally modeled by manual segmentation of the membrane bilayer through sequential tomographic slices in the z-axis of the whole volume with colored membrane contours using either Amira software or the program 3dmod that is part of the IMOD software package [17].

Each compartment in the reconstructed Golgi region was considered a distinct “object,” and a different color was assigned to each object. The portion of each object that was visible in one tomographic slice was traced as a “contour” overlaid on the image. Objects were modeled one at a time. The resolution of the tomogram allowed for most objects to be followed and modeled with complete confidence. After the model completion, a mesh of triangles was then computed to define the surface of each object [17], so that objects could be viewed with standard lighting techniques.

### Immuno-electron microscopy

For immuno-electron microscopy, small samples of the tissue were fixed for 2 h in a mixture of 2% paraformaldehyde and 0.2% glutaraldehyde with 0.1 M phosphate buffer, pH 7.4. Samples were washed with a PBS-glycine solution after fixation. After washing, the samples were embedded in 10% gelatin, cooled in ice and cut into 1 mm^3^ blocks. The blocks were infused with 2.3 sucrose at 4°C overnight and frozen in liquid nitrogen. Ultrathin cryosections (~50 nm) were cut at -120°C with a diamond knife in a Leica Ultracut T/FCS. Cryosections were picked up using a 1:1 mix of 1.8% methylcellulose and 2.3 M sucrose and mounted onto formvar carbon-coated copper grids. The localization of antigens in the cryosections was carried out using the corresponding polyclonal antibody followed by 10 nm protein A-gold [18]. Rabbit anti-mouse was used as secondary antibody for monoclonal antibodies. After labeling, the cryosections were treated with 1% glutaraldehyde, counterstained with uranyl acetate pH 7 and embedded in methyl cellulose-uranyl acetate pH 4 (9:1). Grids were examined with a Jeol-1011 transmission electron microscope.

## Results

### Ultrastructure of the GC of epididymal principal cells

Given the very good preservation of our aldehyde-fixed samples and the difficulties to obtain high quality high-pressure frozen-freeze substitution (HPF-FS) samples, most studies were carried out using chemical fixation (see discussion). As model, we used the principal cells from the initial segment of the epididymis, although the caput region was occasionally photographed as well.

**Fig 1:**
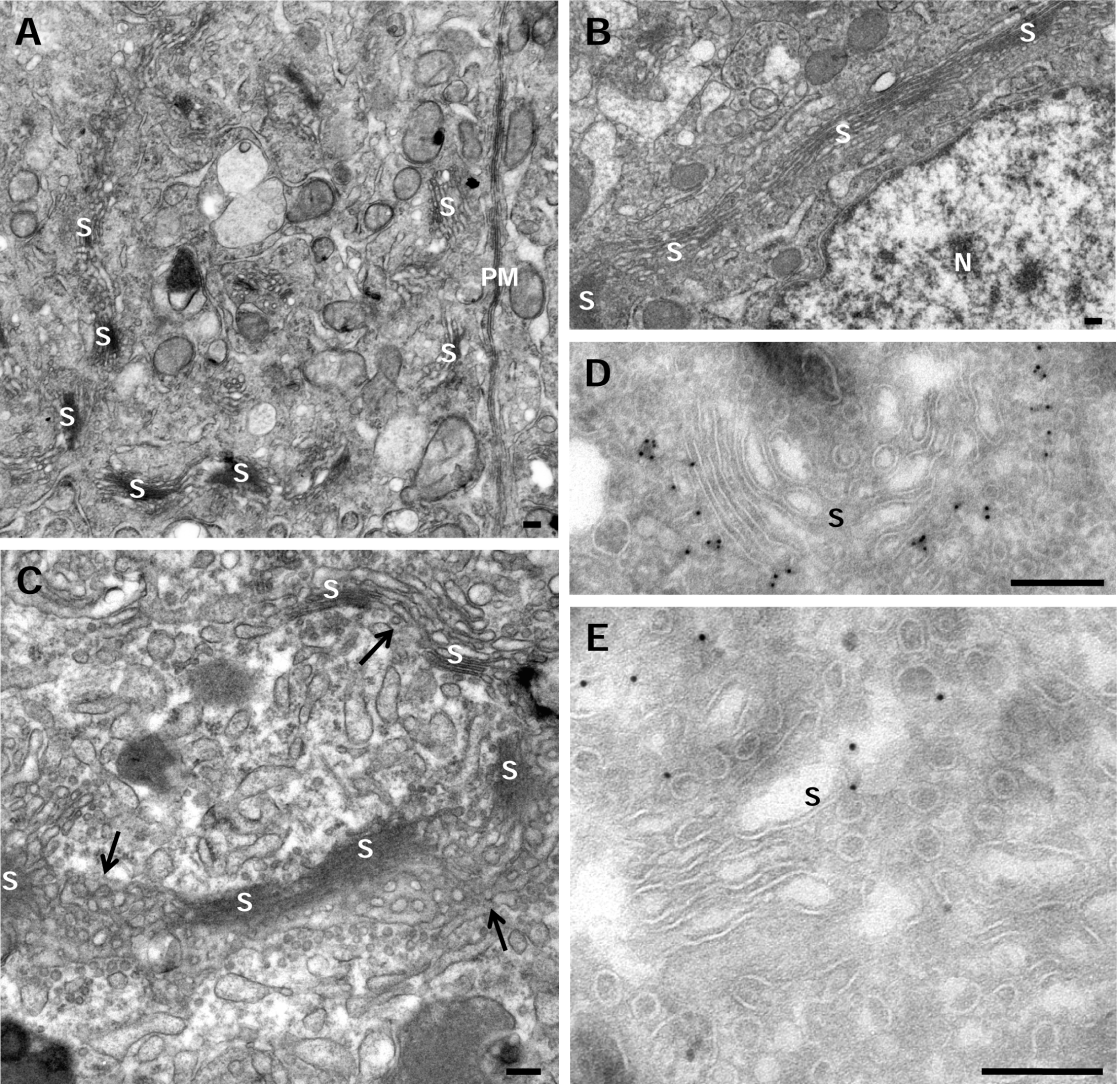
The Golgi complex of epididymal principal cells. **A-C.** Transmission electron microscopy of chemical fixed samples of the initial segment from rat epididymis. **A.** Imaging of this 80 nm ultrathin section shows that the large GC of this cell type is composed of several stacks (S) connected by heteromorphic structures forming a ribbon. Most of these stacks run parallel to the plasma membrane (PM) while others are transversally arranged. **B.** Detail of the stacks (S) adjacent to the nucleus (N) in a 50 nm ultrathin section. **C**. This picture shows the complex nature of elements connecting the stacks (S) formed by heterogeneous membranes representing tubular networks and/or fenestrated cisternae (arrows). **D.** Immunolocation of GM130. This matrix protein is restricted to cis-Golgi elements. **E.** Immunolocation of Rab6. This small GTP is mostly located in tubule-vesicular elements at the trans-Golgi side. Scale bars: 200 nm.

Electron microscopy (EM) analyses of principal epididymal cells in chemically fixed tissue revealed the extraordinary complexity of the Golgi architecture, which had a highly variable appearance. The GC appeared as a very large network composed of branching interconnected cords covering an extensive region of the supranuclear area of the cell. This is especially evident when relatively thick sections (80 nm) where visualized at low magnification (Fig 1A). As shown previously [18, 19], many of the stacks run parallel to the lateral plasma membrane in this region of the epididymis. The GC appeared to be made up of numerous individual stacks separated from each other by large gaps or, conversely, of several stacks connected by heteromorphic structures; it cannot be determined in two dimensions whether they were tubules or highly fenestrated cisternae (Fig 1B and 1C). The complexity of its structure suggests that it is a highly dynamic organelle. Typically a stack is composed of 7–8 cisternae, which are thin or slightly dilated. In addition of morphological criteria (see below), the orientation of the stacks was determined by the immunolocation of GM130 (Fig 1D) and Rab6 (Fig 1E) as cis and trans Golgi markers, respectively.

**Fig 2:**
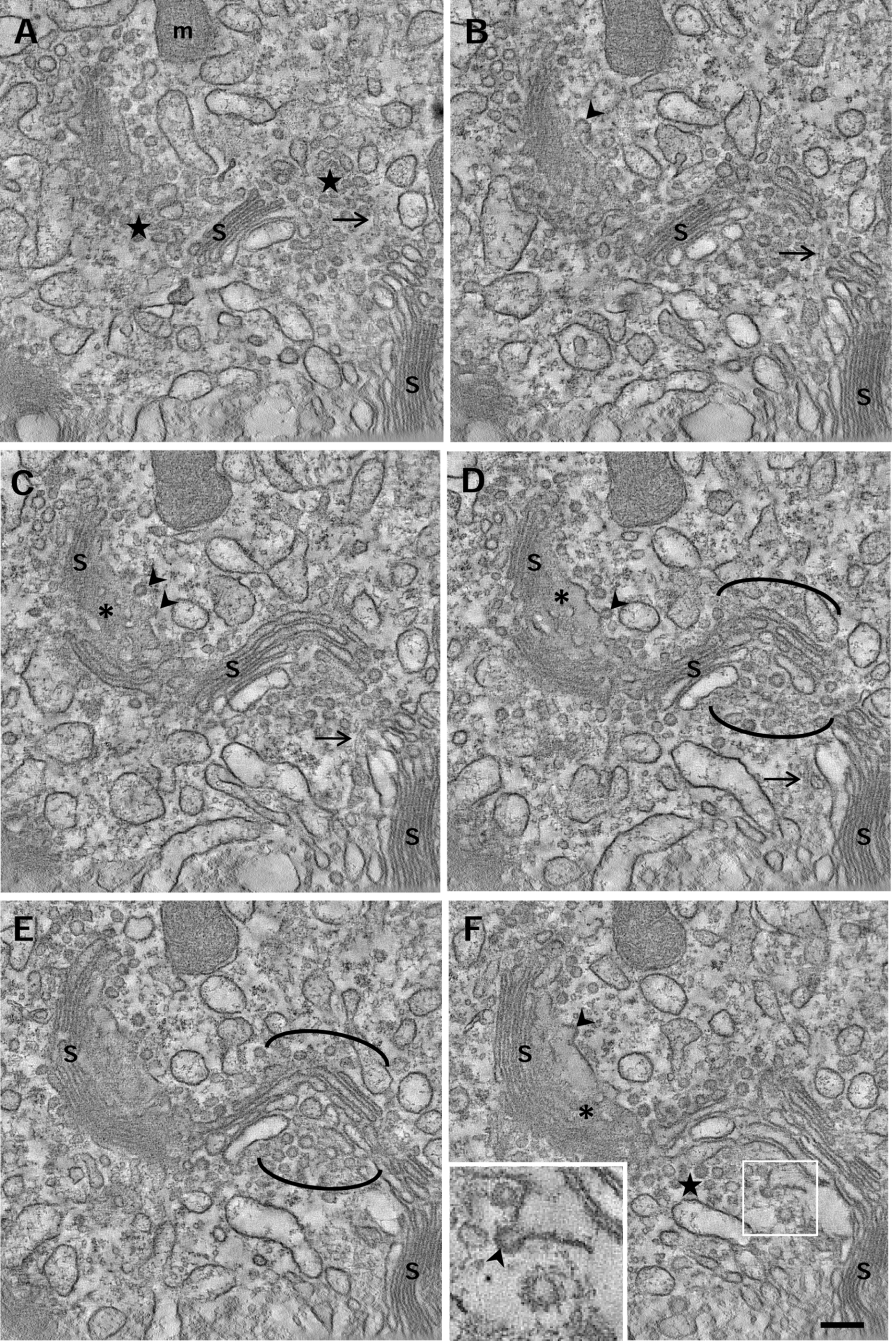
3D overview of the Golgi ribbon. Sequential tomogram slices of a dual-axis tomogram showing an overview of the Golgi area. In Fig A, the GC displays an S-like shape apparently formed by several independent stacks (S), although the analysis of sequential tomogram slices show they were all connected (B-F). The non-compact regions (delimited by parentheses) contain clusters of vesicles (stars) and microtubules (arrows). The inset in Fig F shows a COP-coated bud (arrowhead) close to a clathrin-coated vesicle bearing the characteristic spikes. S, stack; m, mitochondria; asterisks, pores. Volume of the calculated tomogram (x,y,z): 4095 x 4095 x 360 pixels, pixel size 0.59 nm. See also S1 Movie for this tomographic reconstruction.

To examine the complex structure of the Golgi ribbon, more than forty 3D-reconstructions of the perinuclear cytoplasm were performed. The tomograms revealed that apparently independent stacks are actually connected by non-compacted regions forming a continuous ribbon. In fact, all stacks analyzed were connected with other or more stacks suggesting than the entire Golgi complex of the epididymal cells is a unique ribbon. A sequence of z axis slices from a dual-axis tomogram showing an overview of the supranuclear cytoplasm is presented in Fig 2 and in a S1 Movie. The Golgi membranes were highly twisted and displayed an S shape, so even cisternae without apparent bridges may be connected outside the reconstructed volume. The non-compacted regions that laterally connect the stacks (Fig 2, parenthesis) were composed of tubules and/or dilated cisternae and clusters of non-coated vesicles. The complexity and the twisted organization of the Golgi ribbon of principal cells were also appreciated by the fact that it was difficult to identify the cis/trans orientation. However, clathrin- and COP-coated vesicles and buds were unambiguously identified in the tomograms (inset in Fig 2F). In oblique/face views of the cisternae, pores or small fenestrations (Fig 2C, 2D and 2F, asterisks) and buds (Fig 2F, arrowheads) were easily recognized. The quality of the structural preservation as well as the resolution of the tomograms used in this study allowed us to visualize clearly cytoskeletal elements, which are numerous in the Golgi area (see below).

### Structural characteristics of the cisternae

**Fig 3:**
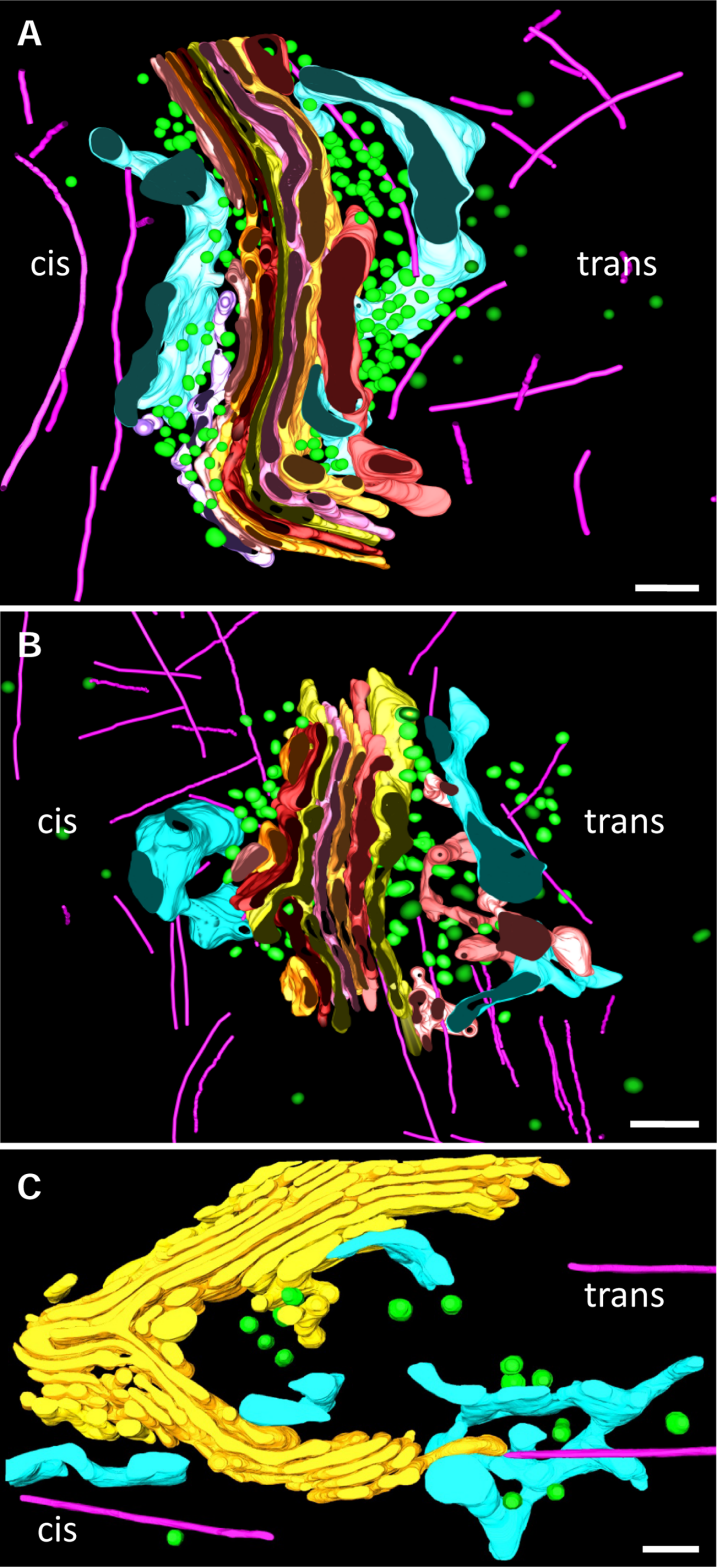
Models in 3D of the GC of principal cells. 3D models generated from dual-axis (A) or single-axis (B,C) tomographic data of the Golgi region. **A,B** Image of the complete model including CGN (light purple), TGN (salmon), cis- and trans-ER (blue), microtubules (fuchsia), small vesicles (green), clathrin-coated vesicles (dark green) and the Golgi stack, whose separated cisternae are in different colors. C1 (light pink), C2 (orange), C3 (dark red), C4 (yellow), C5 (pink), C6 (light orange), C7 (red) C8 (light yellow). **C**. In this model the same colors were used except for cisternae that are displayed in yellow. Volumes of the calculated tomograms (x,y,z): A: 4095 x 4095 x 340 pixels, B: 4095 x 4095 x 336 pixels; C: 4095 x 4095 x 385 pixels; pixel size: 0.59 nm. See also S2, S3 and S4 Movies.

**Fig 4:**
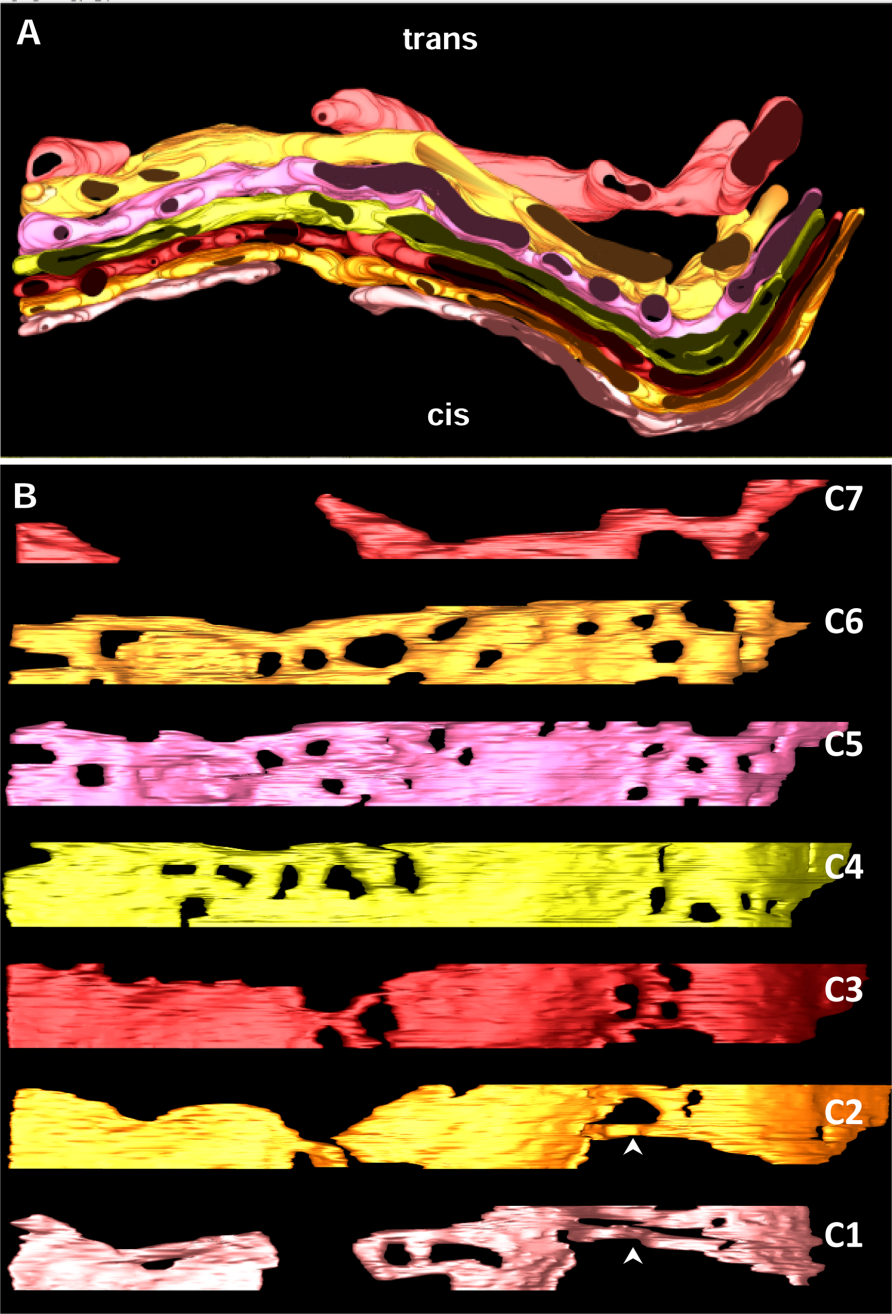
Golgi cisternae. **A.** 3D model generated from Fig 3A by removing all features except the cisternae. **B**. Modeled cisternae are display from cis (C1) to trans side (C7). Arrowheads point to tubular structures at the non-compact region of the ribbon.

In contrast to the complexity of tomographic imaging, segmentation simplifies structure interpretation allowing visualizing and displaying a feature of interest. For that purpose, membranes of the GC, associated tubules and vesicles, ER and microtubules within the tomographic volumes were manually segmented and three 3D-models were performed (Fig 3 and S2 and S3 Movies). The shapes and positions of stacked cisternae and the nearby ER, vesicles, and microtubules were modeled and represented by different colors. When the resulting model was displayed in its entirety, it was so complex that single views were not as informative as one would like. The fine details within the model were masked by all the features that are superimposed. We therefore present our results by disassembling the model. First of all we analyzed the structural features of each cisterna using the GC shown in Fig 3A as model. The segmented cisternae showed a relatively straight region continuous with a more curved one (Fig 4A). The distance between adjacent cisternae was remarkably uniform. The Golgi complex in the reconstructed volume comprised seven cisternae. The individual cisternae were displayed as separate objects and oriented from cis (C1) to trans (C7) sides (Fig 4B). The cis-trans polarity of this Golgi region was deduced from the presence of typical elements of these areas such as the cis-Golgi network, trans-ER and the different types of coated vesicles. Each cisterna had its own structural characteristics. When C1, C2 and C3 were aligned, they are formed by three compact areas connected by two non-compact ones. These non-compact areas were formed by tubular structures establishing homotypical connections (Fig 4B, arrowheads). All cisternae showed small pores somewhere but, when the cisternae were aligned, they did not run parallel from one cisterna to the next. The most fenestrated cisternae were at the trans side, i.e. C5 and C6. Conversely, early cisternae C2, C3 and C4 showed large non-fenestrated regions. The cisterna shape changed progressively from narrow to slightly dilated in the cis-to-trans direction (Fig 4A). In contrast to cisternae C1-C6, C7 had an irregular shape and appeared both detached and fragmented. Based on these morphological features, in addition to the cisternal position, the seven cisternae could be tentatively subdivided into two cis (C1 and C2), two medial (C3 and C4), and two trans (C5 and C6) cisternae, accompanied by one trans-most cisterna (C7) that possesses TGN-like properties. None of these modeled cisternae displayed buds which are usually located at their lateral rims or at the edges of holes.

### Structural evidences of branched cisternae

**Fig 5:**
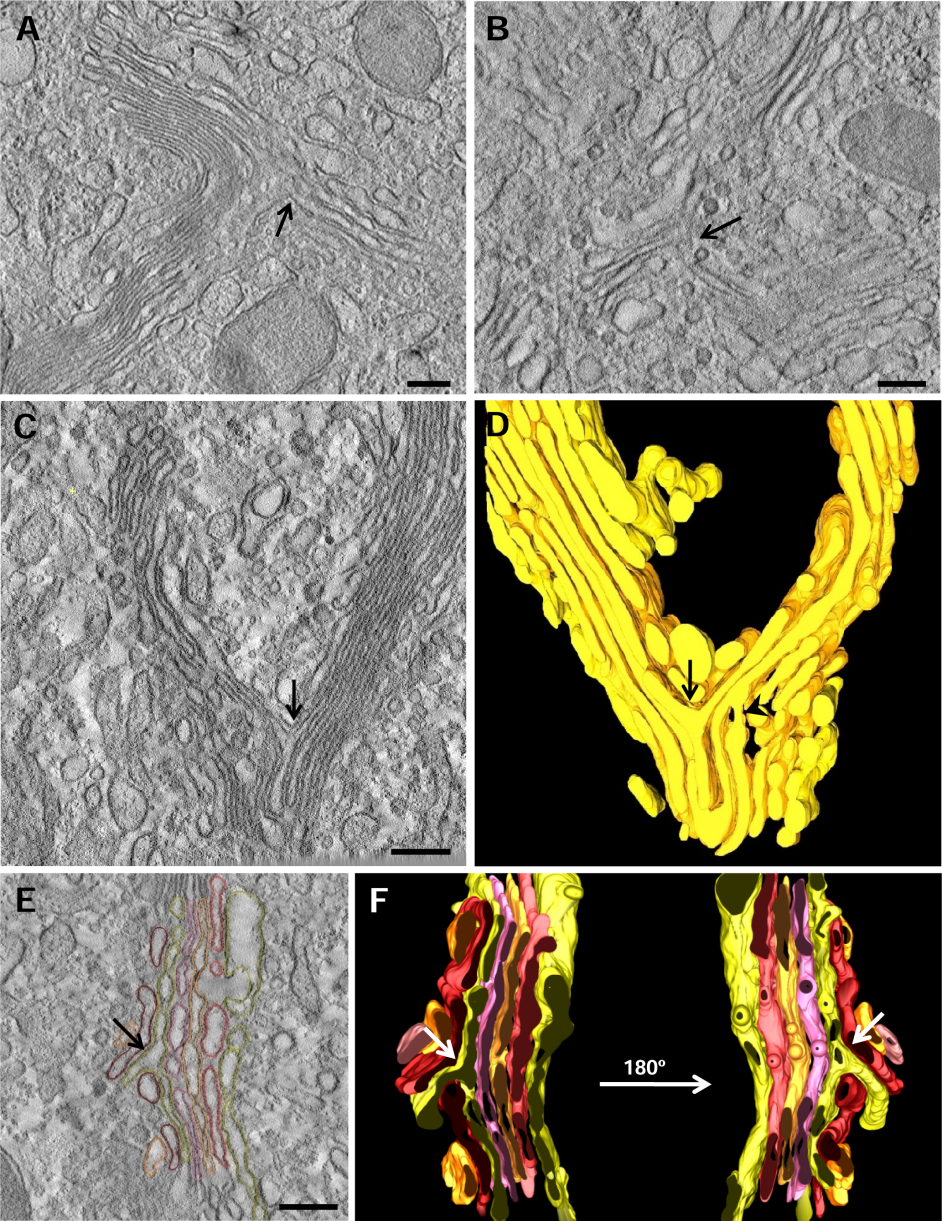
Branched cisternae. **A,B.** Tomographic slices of a portion of the Golgi ribbon from chemically fixed (A) and cryofixed (HPF-FS) (B) samples showing branches (arrows). **C**. Slice of an electron tomographic reconstruction of the stack that shows an Y-shape cisterna (arrow). **D**. Three-dimensional model of the Golgi region shown in C (the complete model is shown in Fig 3C). Apart from the Y-shape cisterna (arrow) there is a connection between two adjacent cisternae (arrowhead). **E**. Slice of a reconstruction of a stack made up of dilated cisternae, where a cisterna projects a branch passing through neighbouring cisternae (arrow). **F**. 3D model of the tomogram reconstruction shown in E (the complete model is shown in Fig 3B). Volumes of the calculated tomograms (x,y,z): A: 4095 x 4095 x 186 pixels, B: 4095 x 4095 x 400 pixels, pixel size 0.59 nm.

As mentioned, the GC of epididymal cells is very complex. We usually observed regions where the Golgi ribbon branches, i.e. places where one stack curves and divides into two or more stacks. The organization and interconnections of the cisternae membranes within these complex and interesting regions were examined in detail. To our initial surprise, we relatively often found branched cisternae (Fig 5A, arrow). Quantitative analysis of tomograms indicated that this structure was found in 8.3 ± 4.7% of the staks (mean ± SEM, n = 36). This unusual feature is not an artifact of chemical fixation because they could be also observed in HPF-FS samples (Fig 5B, arrow). Branches appeared frequently in curved regions of the stack and usually displayed an Y-shape. Occasionally, several Y-shaped cisternae could be found within the same stack. To analyze the complex morphology of these curved stacks, Golgi membranes of a curved stack were traced on the tomographic slices. The 3D-reconstruction and 3D model in Fig 5C and 5D and in S4 Movie showed that cisterna C5 is branched. The three-dimensional analysis demonstrated that this structure is a real branched cisterna and not, for instance, a cisterna and a tubule. Interestingly, in this modeled GC another branched cisterna was observed which seemed to represent a heterotypic connection between adjacent cisternae (Fig 5D, arrowhead). Cisterna branches were also found in straight regions of the stacks. Thus, the Fig 5E and 5F (obtained from model 3B) show that the cisterna C4 projects a branch oriented to the cis direction which passes through openings (fenestrations) in several adjacent cisternae (C1-C3) (arrows).

### Cisterna invaginations

**Fig 6:**
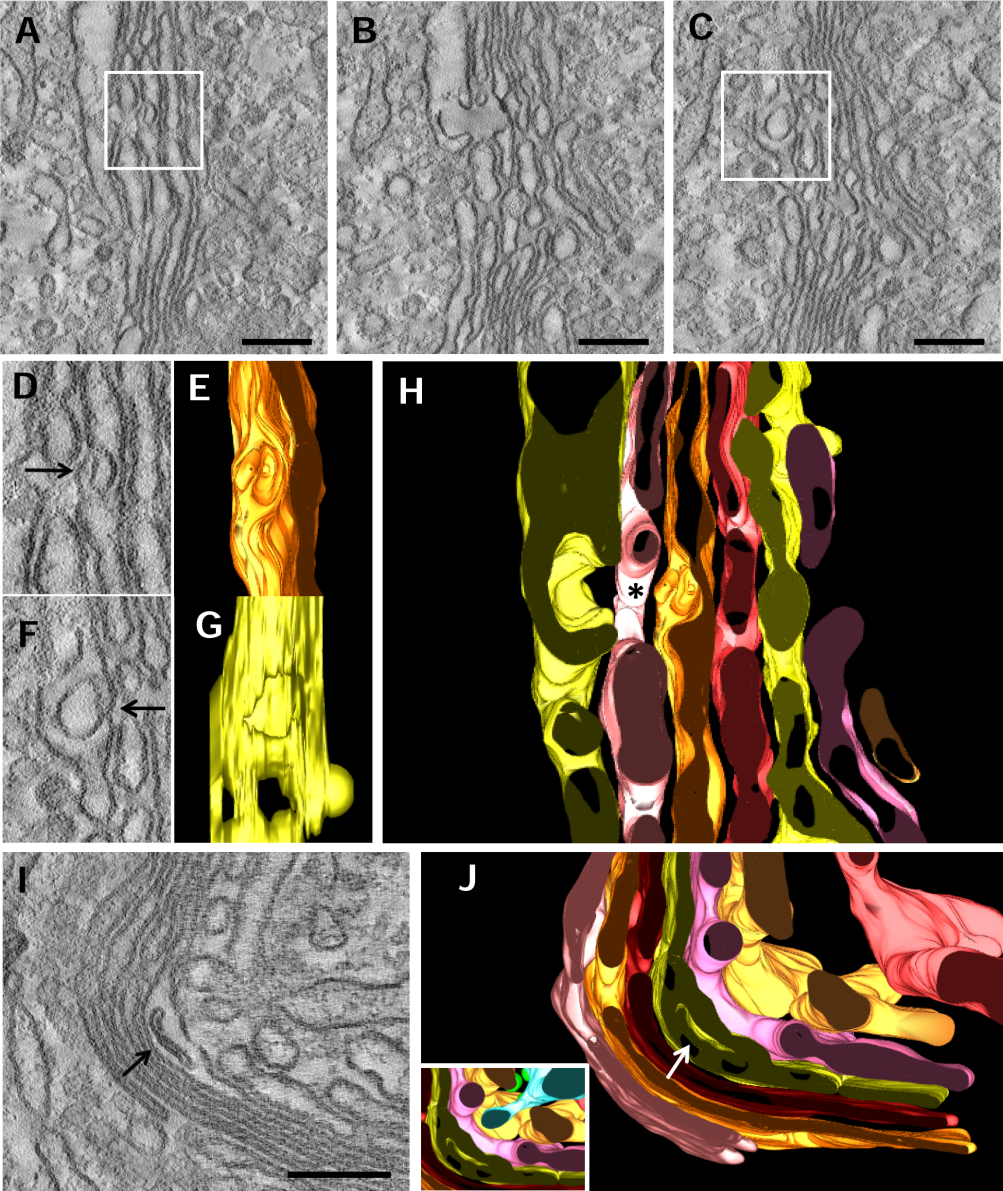
Pocket-like cisterna invaginations. **A-C.** Three sequential slices through the tomogram showing that Golgi membranes are folded forming pocket-like invaginations. The complete 3D model for this GC is shown in Fig 3A. **D.** Detail corresponding to the box in Fig A. **E.** The 3D model in E shows a left side view of the pocket hole shown in D. **F.** Detail of the box in Fig C. **G.** The 3D model shows a right side view of the pocket hole shown in F. **H.** An x/y-plane of the 3D model of the reconstruction showing the inner holes of the pockets (yellow and orange) and the hole in the middle cisterna (pink) are connected forming a corridor (asterisk). The model has the same orientation as the tomographic slices shown in A-C. **I**. Tomographic slice showing a pocket. **J**. 3D model of the reconstruction shown in I. The inset shows an ER tubule (blue) crossing the trans cisternae and approaching to the pocket.

To our initial surprise, 3D reconstruction revealed the presence of cisterna invaginations (Fig 6). These pocket-like structures were found in dilated portions of cisternae and were mainly associated with the trans side. The GC on display in Fig 6A–6H showed cisterna invaginations in both C6 and C8. Reconstructions demonstrated that these structures were open. C7 was interrupted by a pore located at the same level than the pockets on C6 and C8 forming a communication channel between them (Fig 6H, asterisk). Fig 6I and 6J show another example of a cisterna invagination present in a curved region of the stack which is open to the trans side of the stack. Unfortunately, we could not resolve whether this pocket was also open creating a channel across the entire cisterna because the margin of the cisterna was outside the reconstructed volume. An ER tubule was located very close to this pocket (inset in Fig 6J) (described below). Quantitative analysis of tomograms indicated that this structure was found in 13.9 ± 5.9% of the stacks (mean ± SEM, n = 36)

### Endoplasmic reticulum-GC association

**Fig 7:**
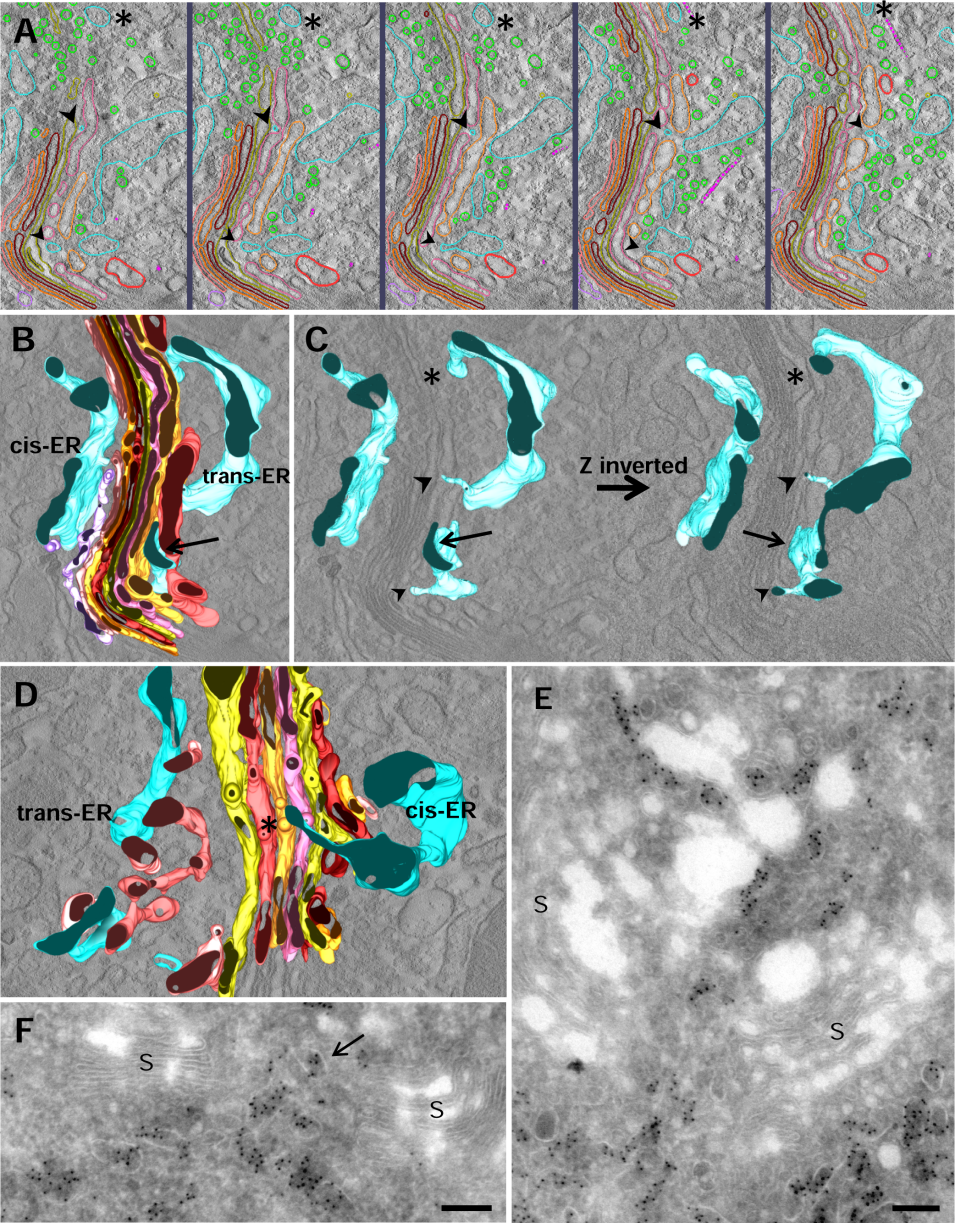
Association endoplasmic reticulum-Golgi complex. **A-C.** Five consecutive tomogram slices (A) and the 3D model corresponding to this tomogram (B, C). ER membranes run parallel to the cis and trans cisternae. Flattened trans-ER lies closely over the trans face of the two trans-most cisternae (arrow). Tubules emerge from the ER inwards, through the trans cisternae (large and small arrowhead). An ER cisterna crosses regions that contain numerous vesicles and tubules and also microtubules (asterisks). **D.** 3D model illustrating an ER tubule (asterisk) crossing a gap (non-compact region) between two stacks at the cis side. **E, F.** Ultrathin cryosections immunolabelled for the ER marker PDI. ER elements surround the stacks (S) at both cis and trans sides but they cross the ribbon through the non-compact regions (arrow). Scale bars: 200 nm.

ER cisternae surrounded cis and trans Golgi sides (Fig 7). At the cis side, ER membranes lied adjacent to both the cis-most Golgi cisterna (Fig 7A–7D) and the discrete polymorphic membranous elements belonging to the ER-Golgi intermediate compartment (ERGIC) (Fig 7A). This cis-ER often showed buds and was surrounded by numerous small vesicles, sometimes arranged in clusters (Fig 7A and 7B). The relationship between the ER and the Golgi cisternae was even more evident at the trans side. As described in other cell types, there was an intimate association between the ER and the trans cisternae of the stack. The extensive trans-ER formed close contacts with the two trans-most cisternae (Fig 7A–7C). The trans-ER wrapped around parts of C7 and was inserted between C6 and C7 (Fig 7B and 7C). These appositions appeared as tight as those between adjacent Golgi cisternae. In addition, we also observed ER-derived tubules emerging from ER inwards the trans cisternae. Fig 7A–7C shows a thin ER tubule (large arrowheads) crossing C5 and C6 and apparently ending in the vicinity of C4. Another ER tubule approaches a cisterna invagination located in C4 after passing the trans-most cisternae (small arrowheads). This specialized trans-ER was continuous with the entire ER network. ER elements were also a common feature of non-compact regions of the stacks (Fig 7D, asterisk). In this figure, an ER cisterna emerging from the cis side crossed these areas filled with small vesicles and tubular elements. The close relationship between ER and Golgi cisternae was confirmed by immunoelectron microscopy using PDI as ER marker. ER was observed all around the stacks (Fig 7E). The presence of ER elements in the non-compact regions of the ribbon was also demonstrated (Fig 7F).

### Relationship between microtubules and the GC

**Fig 8:**
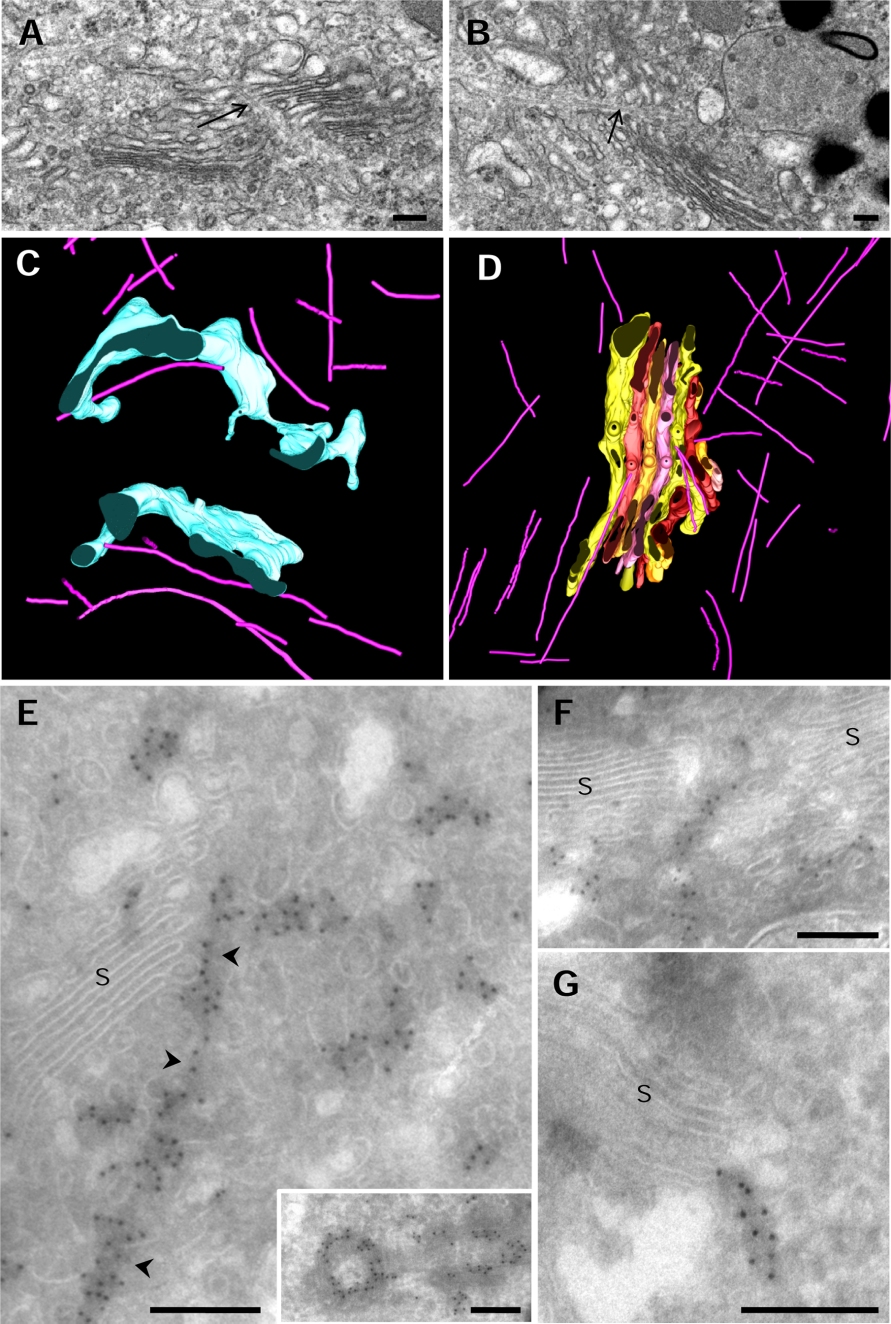
Microtubules and the GC. **A, B**. TEM of chemical fixed principal cells from the epididymis. Micrographs of an ultrathin section show microtubules crossing the Golgi ribbon. **C, D**. 3D models simplified from Fig 3A and 3B, showing the relationship between microtubules and ER (C) and Golgi cisternae (D). **E-G.** Immunolocation of tubulin in ultrathin cryosections. The Golgi area of this cell type shows a high immunoreactivity for tubulin. Arrowheads in Fig E point to a putative microtubule that runs very close to the edge of stack. Tubulin immunoreactivity observed between tubule-vesicular elements may represent cross-sectioned microtubules or soluble tubulin. The inset in Fig E shows a pair of centrioles in the Golgi region of epididymal cells. Immunoreactive microtubules cross the non-compact regions of the ribbon (F) but are also directly associated with cisternae (G). S = stack. Scale bars: 200 nm.

The preservation of membrane integrity after chemical fixation and embedding in epon resins was so good that it enabled us to distinguish the microtubule cytoskeleton in conventional electron microscopy analyses. Microtubules were numerous in the Golgi region of principal cells and were also found traversing the Golgi ribbon at multiple points (Figs 2B–2E, 8A and 8B, arrows). One or two microtubules were observed crossing the non-compact regions of the ribbon confirming that microtubules are common elements. Microtubules crossing the ribbon were also observed in HPF-FS samples (S5 Movie). Electron tomography enabled us to visualize microtubules and follow their trajectory through the reconstructed volume. The 3D models shown in Fig 3A and 3B revealed the relationships between cisternae and the microtubule cytoskeleton. Note that in the modeled regions, microtubules do not exhibit a radial organization. Thus, 8 of the 25 microtubules observed in the tomogram from which Fig 3A was obtained, were parallel to the stack, whereas 13 appeared perpendicular to it. In the next tomogram, 18 of the 40 microtubules, were parallel to cisternae, 14 were perpendicular to them and finally, 3 microtubules were parallel to the branch of C4. In both models, the relationship between the trajectory of ER membranes and the position of microtubules was evident (Fig 8C). Microtubules seemed also associated with densely packed clusters of vesicles (Fig 3A and 3B). Notably, we found microtubules closely associated with cisternae C3, C4 and C7 (Fig 8D, S3 Movie). The association of microtubules and the GC was also analyzed by cryoimmunoelectron microscopy. The affinity and specificity of anti-tubulin antibodies were tested in sperm flagella (not shown) and centrioles (inset in Fig 8E). We found a high immunoreactivity associated with the Golgi area where longitudinal sections of microtubules were often found. Labelling for tubulin was also strong surrounding tubule-vesicular elements indicating the presence of soluble tubulin and/or cross-sectioned microtubules (Fig 8E). Microtubules crossing the ribbon at non-compact regions were detected with this methodology (Fig 8F). As a result of the quality of the antibodies we were able to occasionally find microtubules directly attached to cisternae (Fig 8G).

### Transport intermediates: Vesicles and tubules

Vesicles and tubules are also common elements of the GC of epididymal cells. Vesicles appeared as clusters localized in specific areas between the endoplasmic reticulum and the Golgi cisternae of the cis and trans faces indicating considerable vesicular traffic in the Golgi region of this cell type (Fig 3A and 3B). As described above, vesicles were also found within non-compact regions of the Golgi ribbon (Fig 2). The majority of the population consisted of non-coated vesicles. Most COP-coated vesicles were localized around the cis part of the stacks, whereas clathrin-coated vesicles appeared surrounding the stack but mainly on the trans side. COP-coated buds were also observed at the trans most cisterna adjacent to clathrin vesicles (Fig 2F). In the model presented in Fig 3A, there was a total of 185 vesicles, 14 of them bearing a clathrin coat. A total of 102 vesicles was observed within the Golgi region modeled in Fig 3B, including 17 clathrin-coated vesicles.

**Fig 9:**
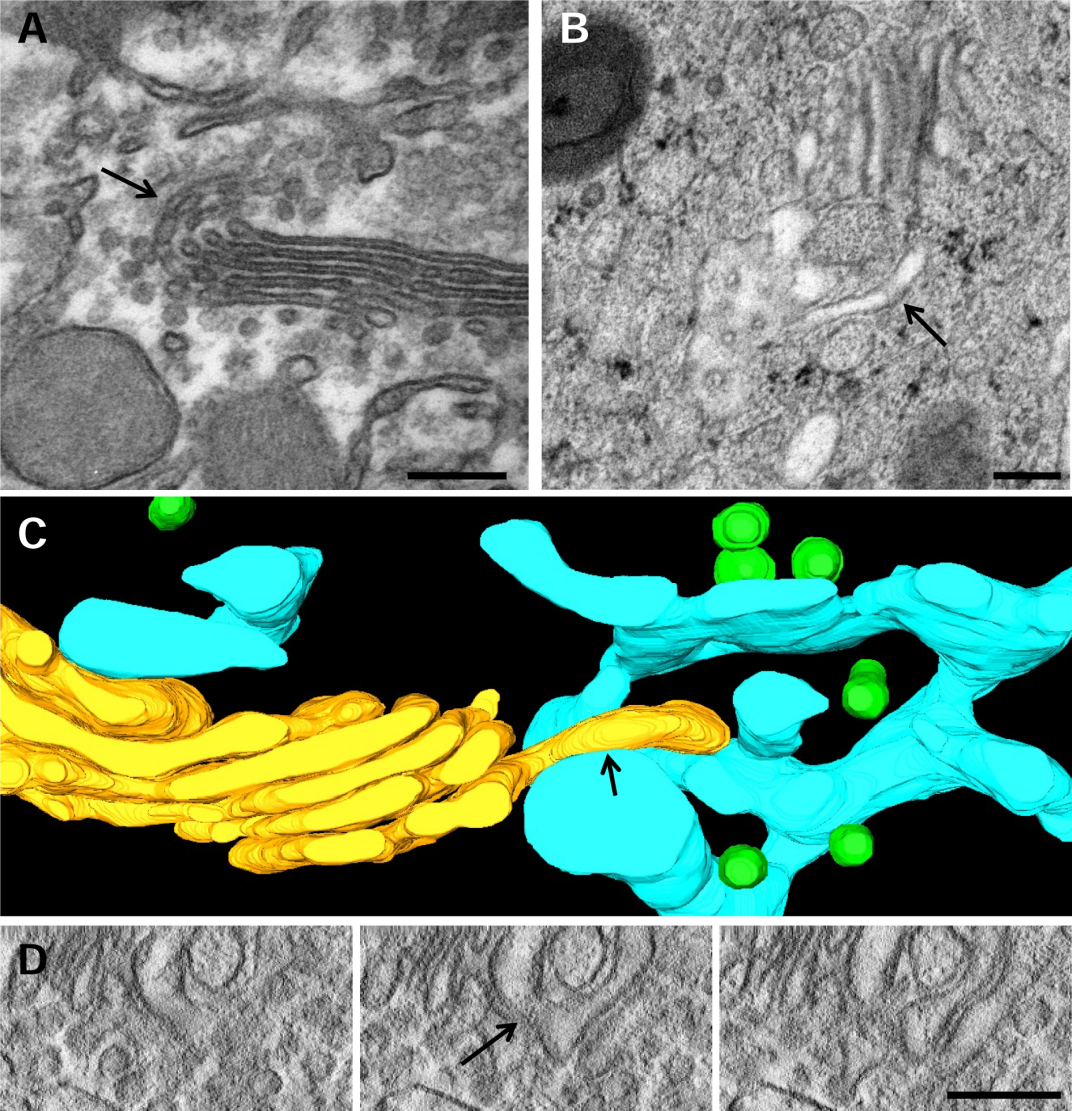
Tubular transport intermediates. **A**. Conventional electron microscopy micrograph showing a tubule (arrow) emerging from the lateral rims of a cisterna. **B**. Face view of a cisterna showing pores and one tubule (arrow) extending outwards, roughly parallel to the plane of the cisterna. **C** 3D-model (obtained from Fig 3C) showing a tubule (arrow) that extends from the edge of the stack into a region surrounded by ER and vesicles. **D**. Sequential z axis slices illustrating a heterotypic connection (arrow) between two cisternae of the same stack. Volume of the calculated tomogram (x,y,z): 4095 x 4095 x 262 pixels, pixel size 0.59 nm. Scale bars: 200 nm.

We also were interested in tubular membranes. These structures are difficult to visualize in two-dimensional images. Electron microscopical analysis of the Golgi apparatus of principal cells showed highly abundant connections between cisternae of tubular appearance (Fig 1C). Examples of Golgi tubules are shown in Fig 9A and 9B. In the first figure, a tubule emerging from the lateral rims of the stack is directed towards the ER (arrow). The second figure shows the face view of a fenestrated cisterna and the associated tubule (arrow). However, in two-dimensional images, these membranes cannot be unambiguously identified as tubules. Tomographic data can be viewed in any orientation in 3D, which allows us to accurately follow tubular profiles. Using this methodology, we unequivocally found tubules extending outwards into the non-compacted region that separate adjacent stacks as shown in Fig 9C (arrow). This tubule grows out from the rim of a cis cisterna and expands through a region surrounded by ER and vesicles (Figs 3C and 9C). Note that this tubule is parallel to the planes of the cisternae and to a microtubule located nearby (Fig 3C). In addition, as shown above (Fig 4B, arrowhead), we found homotypical tubular connections between cisternae. Although it is not a common feature, we also observed tubules connecting two cisternae within the same stack (Fig 9D).

## Discussion

### Preservation of Golgi architecture

The knowledge of the 3D architecture of the GC is prerequisite for evaluating the mechanisms of transit through this organelle. Here we detail the structure of the Golgi ribbon of principal epididymal cells. Most tomographic studies on Golgi organization have been carried out in cryofixed samples of cultured cells. However, it is difficult to obtain a good number of well preserved cryofixed samples of many tissues. Conversely, we got excellent chemically fixed samples of epididymis where the membranes were very well preserved, their tri-laminar organization being apparent, and the cytoskeleton was clearly visible. We used the standard single-axis tomographic approach of semithick (300 nm) sections to compare classical chemical fixation with HPF-FS samples. In some critical cases, we also applied dual-axis tomography in order to obtain better resolution by reducing the loss of information caused by the missing wedge in single-axis tomography. Concerning fixation, we did not find significant differences in the morphology of the membranes of the principal cells with both methodologies in agreement with previous 3D studies of the Golgi area [20]. For that reason most of our studies were carried out with chemically fixed samples, while HPF-FS samples (and immunoelectron microscopy on cryosections) were used to confirm the most important results.

### A 3D view of the GC of principal cells

As mentioned above, the GC of the epididymal cells was extensively explored by Hermo’s group [15, 16]. However, electron microscopy of thin sections selected from multiple cells and/or multiple regions from a single cell cannot reveal the complex organization of this organelle. Electron tomography of well-preserved cells is ideally suited for describing fine ultrastructural details that were not visible with previous techniques. The quality of the structural preservation as well as the resolution of the tomographic “slices” used in this study allowed us to analyze the relationship between the GC (cisternae, vesicles and tubules) and other elements involved in membrane trafficking (i.e., the ER and microtubule cytoskeleton). Furthermore, the magnification of the area reconstructed in this study allows us to visualize fine details of Golgi membranes. So far, most studies about the 3D structure of mammalian GC using this methodology were performed with cultured mammalian cells, where often secretion was stimulated or inhibited [9, 21, 22, 23, 24, 25, 26]. Conversely, we have used cells directly obtained from rat tissues which, under standard conditions, have a very well developed secretory machinery.

The descriptions presented in our study are based on more than 40 tomograms and three models of selected areas of the Golgi ribbon from different principal cells; so we think that they are very representative of the 3D structure of the Golgi complex of principal cells. The main findings are discussed below.

### Branched cisternae

To our knowledge the existence of branched cisternae has not been described previously. This unusual feature is very difficult to observe without a 3D analysis; even in previous tomographic studies it has not been described. In contrast, our 3D-analyses showed that branched cisternae are relatively frequent in epididymal cells. Thus, it is possible that these branches are only present in GC with huge development and highly curved structure but absent or very unusual in other cell types. The branches might be just a method of adaptation of the cisternae to a huge input of secretory material. Moreover, the formation of the branches might be related with the formation of direct tubular continuities between cisternae at different levels of the GC as described in glucose-stimulated mouse islet beta cells [24]. Conversely, it also can be speculated that these structures might be a snapshot of the formation of a new stack within the ribbon.

### Pocket-like cisterna invaginations

Invaginations of the Golgi membranes were a very unusual finding traditionally considered as an artifact of the chemical fixation and/or processing for electron microscopy [11]. However, Bouchet-Marquis and collaborators [27] found these structures in vitreous cryofixed samples, the closest method to native state of the biological material. In our samples, we found several cisternae containing well preserved inner membranes that, after 3D analysis, were found to be all connected to the cytosol, given the appearance of a pocket. Previous analysis and our own data suggest that it is not an artifact and should be taken in consideration as a feature of the Golgi cisternae. In our models, these invaginations were found at the trans side and, occasionally, associated to the trans-ER suggesting that pockets might play a role in lipids transfer by increasing the cisternae surface. We can also speculate that cisterna invaginations might be a mechanism for introducing cytosolic proteins into the secretory pathway which might enter into the cisterna by a process that could resemble microautophagy. This possibility is intriguing and might represent a mechanism for unconventional secretion or, even, degradation. The last possibility cannot be rejected because it has recently been shown that the Golgi complex of neurons is able of engulf cytoplasmatic material for degradation by bending and sealing the stacks [28].

### ER-Golgi association

ER membranes are associated with both cis and trans sides of the stacks. It is well known that, at the cis side, ER is involved in the formation of vesicles as demonstrated by the presence of the characteristic buds. In accordance with previous descriptions in other cell types by electron tomography [21], we have observed an intimate association between the ER and the trans cisternae of the stack. These appositions appear as tight as those between adjacent Golgi cisternae; so that this intimate association may allow direct transfer of lipids such as ceramide and cholesterol between ER and Golgi membranes [9, 29]. In addition, we have observed thin tubules emerging from trans- and cis-ER crossing several trans and cis cisternae, respectively. These ER tubules have been described in cells with stimulated secretion [23] but not in non-stimulated cells [21]. ER tubules are also common elements of the non-compact regions of the ribbon. This finding raises the possibility that ER tubules mediate lipid exchange between the ER and specific Golgi cisternae in highly active secretory cells.

### Microtubules

The structure of the Golgi ribbon and pre- and post-Golgi transport depend on microtubules. Rather than a radial organization, microtubules in the Golgi area of epididymal cells show a cross pattern, with approximately half of the microtubules being oriented parallel to the stack and the other half perpendicular to it. It should be noted that in these epithelial cells from the initial segment of the rat epididymis, most of the stacks run parallel to the lateral membrane whereas others are in a horizontal position and closer to the nucleus so that the Golgi ribbon has a “U” form [19].

The Golgi complex acts as a microtubule-organizing center. Microtubule nucleation at the cis-Golgi depends on AKAP450 which is recruited by the matrix protein GM130 [30]. That could be the case of cis-Golgi associated microtubules found in insulinoma cells [23]. Interestingly, cryoimmunocytochemistry allow us to unambiguously identify microtubules directly connected to cis cisternae. Microtubules can also nucleate at the TGN, a process mediates by CLASP recruited by the TGN protein GCC185 [31]. Interestingly, we observed microtubules associated with the middle/trans cisternae (C3, C4 and C7) so it remains the possibility that middle/trans cisternae can also be places of microtubule nucleation.

In polarized epithelial cells, microtubules are aligned along the apical-basal axis of the cell, with the minus- and plus-ends oriented to the apical and basal membranes, respectively [32]. In addition, there are numerous centrosomal microtubules. In our study microtubules parallel to the stack may represent non-centrosomal microtubules which may be the responsible of the positioning of the ribbon. However, the rest of microtubules may be centrosomal and Golgi-nucleated microtubules which could be involved in transport. As described in an insulinoma cell line, microtubules traverse the ribbon in non-compact regions [23]. However, it is important to note that microtubule organization changes when cells are removed from the tissue and cultured. To our knowledge this is the first study of the 3D relationship of microtubules and GC in tissues.

Microtubules are also involved in ER morphology (i.e. the balance between sheets and tubules), movement and function, including regulation of calcium storage [33, 34]. In agreement with a previous study [23] we found that ER membranes run parallel to microtubules. Some ER membrane proteins bind microtubules. We failed to find these contact points; however, we observed microtubules very close to ER membranes.

## Concluding remarks

Despite of the huge numbers of studies, the GC is still giving surprises. This organelle may have unusual elements in specialized cells, the role of which remains to be clarified. This is the case, for instance, of small (42 nm) clathrin vesicles in neurons [13], TGN associated-COPI vesicles in spermatids [35] and pancreatic cells [36] or branched cisternae and cisternal invaginations in epididymal cells (the present study). The GC features that we have described for the first time in this study are likely related to the huge input of secretory material in these specialized cells. Biochemical, genetics and functional studies must be expanded to more complex cellular models.

## Supporting information

S1 Movie Movie corresponding to the tomographic reconstruction in Fig 2. (M4V)

S2 Movie Movie corresponding to the model presented in Fig 3A. (MP4)

S3 Movie Movie corresponding to the model presented in Fig 3B. (MP4)

S4 Movie Movie of the tomographic reconstruction of the model presented in Fig 3C. (MPG)

S5 Movie Movie of a tomographic reconstruction showing a microtubule going through the Golgi ribbon in HPF-FS samples. (AVI)
